# Birth weight for gestational age norms for a large cohort of infants born to HIV-negative women in Botswana compared with norms for U.S.-born black infants

**DOI:** 10.1186/1471-2431-11-115

**Published:** 2011-12-16

**Authors:** Lynn T Matthews, Heather J Ribaudo, Natasha K Parekh, Jennifer Y Chen, Kelebogile Binda, Anthony Ogwu, Joseph Makhema, Sajini Souda, Shahin Lockman, Max Essex, Roger L Shapiro

**Affiliations:** 1 Beth Israel Deaconess Medical Center, Division of Infectious Disease, 110 Francis Street, Lowry Medical Office Building - Suite GB, Boston, MA 02115, USA; 2 Massachusetts General Hospital, Division of Infectious Disease, Center for Global Health, 100 Cambridge Street, Boston, MA, 02114, USA; 3 Harvard School of Public Health, 651 Huntington Avenue, FXB Building - Room 509, Boston, MA 02115, USA; 4 University of Miami, Miller School of Medicine, 1600 NW Tenth Avenue, Miami, FL 33136, USA; 5 Harvard Medical School, 25 Shattuck Street, Boston, MA 02115, USA; 6 Botswana Harvard AIDS Institute, Private Bag BO 320, Bontleng, Gaborone, Botswana; 7 Brigham and Women's Hospital, Division of Infectious Disease, 75 Francis Street, Boston, MA 02115, USA; 8 Harvard School of Public Health, Departments of Immunology and Infectious Disease, 651 Huntington Avenue, FXB Building - Room 401, Boston, MA 02115

## Abstract

**Background:**

Standard values for birth weight by gestational age are not available for sub-Saharan Africa, but are needed to evaluate incidence and risk factors for intrauterine growth retardation in settings where HIV, antiretrovirals, and other *in utero *exposures may impact birth outcomes.

**Methods:**

Birth weight data were collected from six hospitals in Botswana. Infants born to HIV-negative women between 26-44 weeks gestation were analyzed to construct birth weight for gestational age charts. These data were compared with published norms for black infants in the United States.

**Results:**

During a 29 month period from 2007-2010, birth records were reviewed in real-time from 6 hospitals and clinics in Botswana. Of these, 11,753 live infants born to HIV-negative women were included in the analysis. The median gestational age at birth was 39 weeks (1^st ^quartile 38, 3^rd ^quartile 40 weeks), and the median birth weight was 3100 grams (1^st ^quartile 2800, 3^rd ^quartile 3400 grams). We constructed estimated percentile curves for birth weight by gestational age which demonstrate increasing slope during the third trimester and leveling off beyond 40 weeks. Compared with black infants in the United States, Botswana-born infants had lower median birth weight for gestational age from weeks 37 through 42 (p < .02).

**Conclusions:**

We present birth weight for gestational age norms for Botswana, which are lower at term than norms for black infants in the United States. These findings suggest the importance of regional birth weight norms to identify and define risk factors for higher risk births. These data serve as a reference for Botswana, may apply to southern Africa, and may help to identify infants at risk for perinatal complications and inform comparisons among infants exposed to HIV and antiretrovirals *in utero*.

## Background

Each year over 4 million infants die in the first four weeks of life (the neonatal period). Ninety-eight percent of neonatal deaths take place in the developing world, and the highest risk is in Africa, where an average of 41 neonatal deaths occur per 1000 live births [1]. Botswana, a middle-income country in southern Africa, has a well-developed medical infrastructure where 97% of women access antenatal care, 94% of births are overseen by a skilled attendant, and 80% of women access hospital-based obstetrical care for deliveries [1]. However, neonatal mortality in Botswana remains high, estimated at 46/1000 live births in 2004 [1].

Botswana is in the midst of a generalized HIV epidemic, and up to a third of infants are born to HIV-infected women [2]. HIV infection is associated with adverse birth outcomes and early infant mortality [3-12]. Although the use of combination antiretroviral drugs for maternal health and for the prevention of mother-to-child HIV transmission is likely to reduce overall infant mortality by decreasing HIV infection among infants [13-15], the use of antiretrovirals (ARVs) has also been associated with lower birth weights [16-19]. A better understanding of the links between HIV, ARVs, and birth outcomes is required, particularly in resource-limited settings where obstetric and pediatric resources are often limited [17].

Low birth weight infants (< 2500 grams) are at risk for early death [20,21]. Weight by gestational age is an important outcome that controls for effects of prematurity and is interpreted as a proxy for intrauterine growth restriction [20,22-25]. Small for gestational age infants (birth weight < 10^th ^percentile for gestational age) are at risk for complications such as peripartum asphyxia, birth trauma, hypoglycemia, impaired neurological development and perinatal mortality [26-30]. Because birth weights may vary regionally, the creation of specific norms for birth weight by gestational age in Botswana may be an important step towards identifying infants at risk for early death [27,31,32].

In this report we describe birth weight for gestational age for a large cohort of infants born to HIV-negative women in 6 hospitals in Botswana. Botswana is well-suited for development of these norms as the majority of births occur in hospital settings where information on birth weight, gestational age, and maternal HIV status is available. We compare these data with U.S. birth weight data for black infants with the goal of providing reference data for assessing birth weight for gestational age for both HIV-exposed and unexposed infants in Botswana.

## Methods

### Study population

Birth weight and gestational age were recorded for live births at six government facilities over a 29 month period from October 19, 2007 to March 16, 2010. Surveillance started at Princess Marina Hospital in Gaborone, the largest hospital in Botswana. Surveillance began in 2008 at Scottish Livingstone Hospital in Molepole and at Broadhurst and Old Naledi clinics in Gaborone. In 2009, surveillance expanded to Deborah Reteif Hospital in the village of Mochudi in southern Botswana, Ghanzi Primary Hospital in western Botswana, Letsolathebe Hospital in Maun in northwestern Botswana, and to Nyangabgwe Hospital in Francistown, the largest city in northern Botswana.

Princess Marina Hospital is the largest hospital in Botswana and serves as a tertiary referral center for the southern part of the country; the Broadhurst and Old Naledi clinics are smaller clinics in Gaborone. Scottish Livingstone Hospital and Debora Reteif hospitals are district-level referral centers. The hospitals in Francistown and Ghanzi serve as referral centers for northern and western Botswana, respectively.

This study was approved by the Office of Human Research Administration at the Harvard School of Public Health, by the Health Research Development Committee in Botswana, and by all participating hospitals.

### Outcomes and Exclusions

Birth weights were abstracted (JYC, NP) from obstetric records and delivery registries completed by maternity nurses. Extracted data included birth weight, infant gender, delivery date, gestational age, maternal age, and documented HIV status. In the event of multiple gestations, the outcome of the first-born infant was recorded. Clinical staff assessed and recorded gestational age at delivery using last normal menstrual period and fundal height assessment. Gestational ages are not confirmed by ultrasound in Botswana's public hospitals.

**Figure 1:**
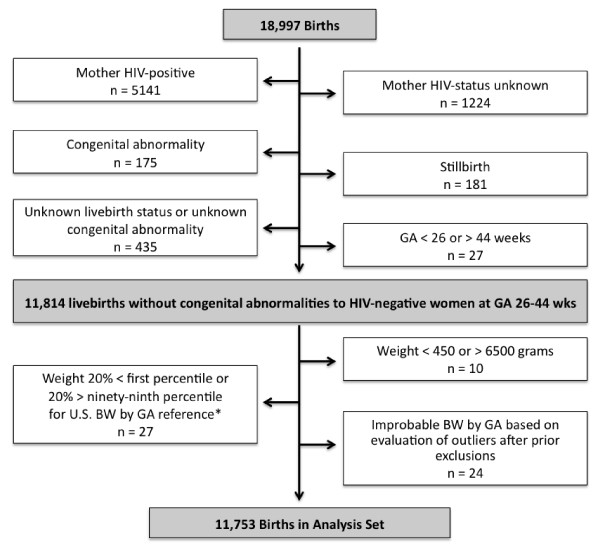
**Selection of analysis dataset**. Analysis set limited to live births without congenital abnormalities born to HIV-negative women; outliers removed as discussed in methods. * Oken E, Kleinman KP, Rich-Edwards J, Gillman MW. A nearly continuous measure of birth weight for gestational age using a United States national reference. BMC Pediatrics 2003;3:10.

Our analyses included live births to HIV-negative mothers between 26 and 44 weeks of gestation with no known congenital abnormality. Cases for which a live birth could not be confirmed (including still-births and those of unknown status) and births to women of positive or unknown HIV-status were excluded. Births with documented congenital abnormalities were also excluded. Because of concerns regarding data accuracy, infants with recorded birth weight less than 450 grams or greater than 6500 grams across all gestational ages and those with a recorded birth-weight that was 20% lower than the first percentile or 20% higher than the ninety-ninth percentile of published distributional norms of black infants born in the U.S. were also excluded [25]. This U.S. reference represents a large, comprehensive dataset and detailed data were available in the public domain. Outlier weights identified in the prior step were reviewed by four pediatricians. Values that the majority judged to be reasonable to exclude or include were treated accordingly [32]. (See Figure 1.)

### Statistical Methods

Smoothed birth weight percentiles for gestational ages 26-44 weeks were estimated using quantile regression analyses with natural cubic B-spline with knots at weeks 24, 26, 28, 38, 40, 44 [33-35]. Knot selection was determined based on visual inspection of the fit and distributional shape compared to published birth weight data from other settings [31,36-38]. Estimated percentile curves at gestational ages less than 30 or greater than 43 weeks are not plotted but the data for these extremes of gestational age were retained in the analysis for modeling purposes. Use of this broader data range in modeling provides a more realistic representation of growth profiles over time. However, the small sample size and, thus, estimate precision limits the generalizability at these data extremes. All summary data are presented in Table 1.

**Table 1:** Distribution of birth weights for gestational age for babies born to HIV-negative women in Botswana compared with median for babies born to black women in the U.S.

Gestational Age (weeks)	N	Minimum (grams)	Lower Quartile (grams)	Median (grams)	Upper Quartile (grams)	Maximum (grams)	U.S. median * (grams)	Δ Median (Botswana - U.S.)	**p-value**^**†**^
26	9	700	880	930	1230	1680	857	73	.051
27	20	670	1013	1116	1610	2310	977	139	.004
28	29	790	1120	1260	1740	2700	1120	140	.003
29	54	750	1200	1375	1725	2950	1304	71	.015
30	62	860	1390	1660	2180	2990	1525	135	.002
31	71	810	1535	1815	2630	3300	1804	11	.032
32	119	800	1680	2085	2730	3440	2084	0.6	.099
33	162	1170	2055	2410	2790	3790	2358	52	.370
34	268	1380	2100	2510	2928	3850	2571	- 61	.095
35	394	1180	2320	2700	3060	4200	2733	- 33	.172
36	687	1470	2560	2850	3115	4335	2870	- 20	.164
37	1056	1790	2690	2960	3240	4680	3014	- 53	< .001
38	1758	1900	2820	3060	3330	4550	3158	- 98	< .001
39	2340	1890	2890	3130	3405	5150	3277	- 147	< .001
40	2482	1960	2970	3220	3500	4920	3356	- 135	< .001
41	1357	1980	3000	3290	3600	5000	3399	- 109	< .001
42	746	2000	3000	3300	3610	5080	3346	- 45	.016
43	125	2300	3000	3280	3540	4405	3316	- 35	.492
44	14	2680	3100	3345	3700	4785	3328	16	.583

* Oken E, Kleinman KP, Rich-Edwards J, Gillman MW. A nearly continuous measure of birth weight for gestational age using a United States national reference. BMC Pediatrics 2003;3:10.

**^† ^**Wilcoxon signed rank test.

Wilcoxon rank sum test, stratified by gestational age, was used to compare birthweight for gestational age among girls and boys. Birthweight at each gestational age was compared with the U.S. median weight for that gestational age using Wilcoxon signed rank test. All analyses were performed in SAS 9.2 for UNIX.

## Results

The initial dataset (18,997 births) was first restricted to 11,814 live births, born to HIV-negative women, without congenital abnormality, between 26 and 44 weeks of gestation. An HIV status was recorded for 94% of mothers in the initial dataset. Of the excluded outcomes, 5,141 (72%) were births to HIV-positive women and 1,224 were to women of unknown HIV status. Among the excluded births to HIV-negative women, 181 were stillbirths, 175 had a congenital abnormality, 435 had unrecorded birth or congenital abnormality outcome, and 27 had gestational age less than 26 or greater than 44 weeks. In addition, sixty-one outliers were removed as described in the methods, leaving a total of 11,753 in the analysis dataset (Figure 1). For the final analysis dataset, 8,009 (68%) of captured births were from Princess Marina Hospital, 1,563 (13%) from Scottish Livingstone Hospital, 763 (8%) from Nyangabgwe (Francistown), while < 5% (700 or fewer) came from each of the remaining sites.

Data for weight by gestational age ranging from 26 to 44 weeks are shown in Table 1. The number of infants at each gestational age ranged from 9 at 26 weeks to 2482 at 40 weeks. Among all infants, the median gestational age at the time of birth was 39 weeks, and the median birth weight was 3100 (IQR 2800-3400) grams. Boys were generally heavier than girls at each gestational age with a median of 3160 (IQR 2850, 3480) grams compared with 3030 (IQR 2750, 3325) grams (p < .0001), respectively.

**Figure 2:**
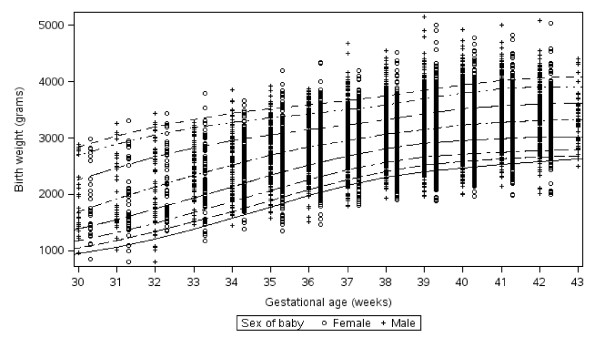
**Percentile curves for birth weights among babies born to HIV-uninfected women in Botswana, by gestational age**. Percentiles using a B-spline with knots at 24, 26,28,38,40,44 weeks. Actual birth weight data plotted.

Figure 2 shows estimated percentile curves from 30-43 weeks gestation for 11,627 males and females combined from the final analysis dataset. These curves are similar to prior birth weight for gestational age data with increasing slope during the third trimester and leveling off beyond 40 weeks [25,39].

**Figure 3:**
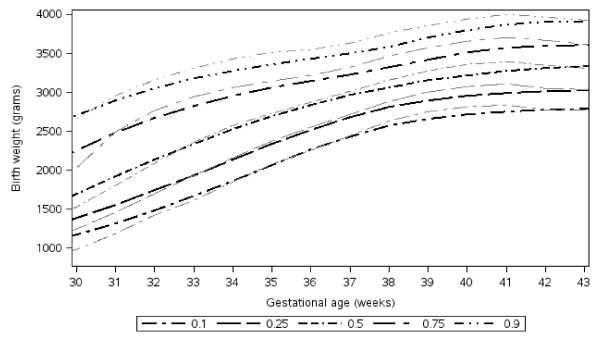
**Percentile curves for Botswana- compared to U.S.- born black babies**. Percentiles using a B-spline with knots at 24, 26,28,38,40,44 weeks (black); published percentiles for black U.S.-born babies shown in gray*. * Oken E, Kleinman KP, Rich-Edwards J, Gillman MW. A nearly continuous measure of birth weight for gestational age using a United States national reference. BMC Pediatrics 2003;3:10.

Figure 3 shows percentile curves constructed from the Botswana dataset superimposed with curves for U.S.-born black infants [25]. Observation of the curves and the data (Table 1) suggest that Botswana-born infants tended to be larger than U.S.-born infants after shorter gestation (< 34 weeks) and smaller than U.S. born infants after longer gestation (≥ 34 weeks). These observed differences are significant at weeks 27-31 and weeks 37-42 (p ≤ .04, Table 1).

Table 2 shows weight by percentile for gestational age. The 10^th ^percentile for U.S. data is shown for comparison. The 10^th ^percentile (the standard cut-off for small for gestational age) values are observed to be higher for pre-term babies in Botswana than for pre-term U.S.-born babies, but to be similar at and beyond term.

**Table 2:** Estimated birth weight percentile by gestational age for babies born to HIV-negative women in Botswana

		Estimated birth weight percentile (grams) by gestational age								
**Gestation age (weeks)**	**N**	**3**^**rd**^	**5**^**th**^	**10**^**th**^	**25**^**th**^	**50**^**th**^	**75**^**th**^	**90**^**th**^	**95**^**th**^	10^th ^U.S.*

30	62	946	1042	**1170**	1380	1693	2255	2699	2840	**980**
31	71	1067	1179	**1318**	1553	1915	2475	2895	3038	**1188**
32	119	1214	1336	**1485**	1740	2130	2664	3054	3200	**1419**
33	162	1384	1509	**1670**	1936	2333	2822	3179	3330	**1616**
34	268	1574	1697	**1866**	2135	2520	2950	3277	3433	**1849**
35	394	1777	1891	**2067**	2332	2688	3055	3356	3517	**2070**
36	687	1979	2082	**2260**	2517	2834	3146	3427	3590	**2248**
37	1056	2165	2258	**2433**	2680	2960	3230	3500	3663	**2439**
38	1758	2313	2405	**2570**	2810	3065	3320	3588	3747	**2632**
39	2340	2409	2510	**2660**	2900	3152	3420	3697	3848	**2750**
40	2482	2471	2580	**2714**	2957	3220	3511	3800	3945	**2814**
41	1357	2525	2626	**2750**	2992	3270	3570	3867	4015	**2836**
42	746	2577	2654	**2773**	3012	3305	3600	3900	4060	**2779**
43	125	2629	2670	**2788**	3021	3330	3610	3910	4088	**2770**

* 10^th ^percentile for U.S.-born black infants. Oken E, Kleinman KP, Rich-Edwards J, Gillman MW. A nearly continuous measure of birth weight for gestational age using a United States national reference. BMC Pediatrics 2003;3:10

## Discussion

This report describes infant birth weight for gestational age for the largest cohort of infants born to HIV-negative women reported from southern Africa. These data may serve as a reference for identifying infants at risk for early complications and for assessing potential risk factors for adverse infant outcomes, including maternal HIV infection and exposure to antiretrovirals *in utero*.

These data may be representative for births in Botswana. Based on annual and estimated birth rates for Botswana, there were 114,620 births over the 29 months of surveillance [40]. The initial data set included 18,997 or about 17% of recorded births for the period. In addition, 10.8% of infants in this dataset meet the definition for low birth weight which is consistent with national statistics reporting 10% incidence [20]. Although Princess Marina Hospital, the largest hospital in the country, contributed the majority of the data, the additional sample sites were located throughout the country and included primary sites as well as regional and national referral centers. Because an estimated 80% of births occur in healthcare facilities, institutional deliveries should approximate population norms. Those 20% of births that were not captured may represent a combination of rural, poorer women who cannot readily access healthcare as well as more affluent women who access private clinics.

Birth weight monitoring is complicated in countries where the majority of births occur outside healthcare facilities. For most developing countries, birth weight data are collected via annual Demographic Health Survey records and birth weight for gestational age is generally unavailable [20,41]. Institutional birth weight data from Botswana offer a more reliable representation of population norms compared to many other developing country settings since 80% of women access hospital-based obstetrical and neonatal care [1]. Data from hospital-based deliveries in Botswana may therefore be useful to other countries in southern Africa.

The Botswana-born infants had higher average birth weights pre-term (statistically significant at 27-31 weeks) and lower birth weights at term (statistically significant at 37-42 weeks) than U.S.-born infants in the referent dataset. Several studies have explored the potential inaccuracies of gestational age dating by last menstrual period. Dating is often inaccurate due to recall bias, variable menstrual cycles, and misinterpretation of bleeding at the time of embroyo implantation. The summation of these errors results in underestimation of prematurity (thus higher birth weights pre-term) and overestimation of post-dates (thus lower birth weights post-term) [42-46]. Distributions around term tend to be less affected by this variation, in part due to larger sample sizes. Among women in the analysis dataset who had antenatal clinic visit information (~50%), 98% had attended an antenatal clinic at least once, which may increase the reliability of the dating. Both datasets estimated gestation age based on last menstrual period, but it is likely that the larger numbers in the U.S. dataset improved precision. Our sample sizes exceeded 100 between 32-41 weeks and thus we have the most confidence in these numbers. It is also possible that Botswana-born children have lower birth weight at term than U.S.-born black infants due to ethnic and/or racial variability of third trimester birth weight for gestational age [25,27,32] or due to environmental differences.

Because Botswana-born term babies were smaller than U.S.-born babies, the 10^th ^percentile cut-offs were lower. By convention, the 10^th ^percentile is the cut-off to define small for gestational age [47]. Additional research will be required to assess whether infants at highest risk are those below the 10^th ^percentile or if in this setting a larger proportion of infants are at risk for early mortality.

There are several limitations to these data. Reported data were collected from six different hospitals, however 68% of the data were from Princess Marina Hospital, a tertiary referral site. These women may represent a wealthier urban population, as well as women referred with complicated pregnancies. In addition, these data were not restricted to singleton births, thus potentially skewing the data towards lower birth weights. Third, estimated gestational age may be inaccurate when estimated from last menstrual period [42,45]. We attempted to correct the data by excluding implausible birth weights at each gestational age, and by restricting the dataset to include only infants from 26 to 44 weeks gestation. Multiple strategies for exclusion of implausible birth weights have been explored [32,36,39,48-51] and several strategies were applied to these data. The method we employed resulted in the best fit for constructed growth curves. In addition, the comparison dataset was taken from published norms for black infants born in the U.S; however, birth weights by gestational age for black U.S.-born children are lower than for other U.S.-born groups [25,39]. In addition, the reference dataset includes HIV-positive and HIV-negative mothers. Given that the HIV-prevalence for black women in the U.S. is estimated at 1.1%, this is unlikely to significantly impact the data [52]. Finally, we only included data for women who had a known negative HIV status; this limits population-wide applicability, but will facilitate future comparisons of HIV-exposed infants with the norms presented in this dataset. This group will publish the findings for infants born to the HIV-positive women in a separate manuscript [19].

## Conclusions

We present a reference for birth weight for gestational age for a large sample of infants born to HIV-negative women in hospitals in Botswana, and demonstrate lower median term birth weights than reported for black infants born in the United States. These data should prove useful for future research investigating the determinants of neonatal mortality and for assessing the effects of HIV and antiretrovirals on infant birth weight.

## List of abbreviations

HIV: Human immunodeficiency virus; ARVs: antiretrovirals.

## Competing interests

The authors declare that they have no competing interests.

## Authors' contributions

LTM was responsible for analysis, presentation and primary authorship of the work presented here. HR was primarily responsible for conception, design, analysis, interpretation and contributed to writing. NP and JYC acquired the data; KB contributed data collection and analysis. AO, JM, SS, SL, ME were involved in conception and study design. RLS was involved in all phases of the work. All authors reviewed and approved the final manuscript.

## Pre-publication history

The pre-publication history for this paper can be accessed here:

http://www.biomedcentral.com/1471-2431/11/115/prepub
